# Effect of Feldspar Substitution by Basalt on Pyroplastic Behaviour of Porcelain Tile Composition

**DOI:** 10.3390/ma14143990

**Published:** 2021-07-16

**Authors:** Mateus Locks, Sabrina Arcaro, Carlos P. Bergmann, Manuel J. Ribeiro, Fabiano Raupp-Pereira, Oscar R. K. Montedo

**Affiliations:** 1 Programa de Pós-graduação em Ciência e Engenharia de Materiais–PPGGEM, Laboratório de Cerâmica Técnica–CerTec, Universidade do Extremo Sul Catarinense–UNESC, Criciúma 88806-000, SC, Brazil; mateus@gruposetep.com.br (M.L.); sarcaro@unesc.net (S.A.); fraupp@unesc.net (F.R.-P.); 2 Graduate Program in Mining, Metallurgical and Materials Engineering–PPGE3M, Federal University of Rio Grande do Sul–UFRGS, Porto Alegre 90040-060, RS, Brazil; bergmann@ufrgs.br; 3 Materials Research and Development Center (UIDM), Polytechnic Institute of Viana do Castelo (IPVC), Rua Escola Industrial e Comercial de Nun’Álvares, 4900-347 Viana do Castelo, Portugal; ribeiro@estg.ipvc.pt

**Keywords:** basalt, porcelain tile composition, optical fleximetry

## Abstract

This work aims to evaluate the effects of feldspar substitution by basalt on porcelain tile composition with respect to its porosity, flexural strength, and pyroplastic deformation. Three ceramic formulations with different amounts of feldspar substituted with basalt, 50% (C1), 75% (C2), and 100% (C3), were evaluated at three different temperatures, 1200, 1220, and 1240 °C. Specifically, the effect of replacing feldspar with basalt on the pyroplastic deformation of ceramic bodies was analysed using optical fleximetry. The porosity of C1 at 1200 °C was 19.3 ± 2.9%, while that of composition C3 was 22.2 ± 0.7% at 1240 °C. The flexural strength was strongly influenced by the temperature. For C1 at 1200 and 1240 °C, flexural strengths of 11.1 ± 0.6 and 22.2 ± 1.9 MPa, respectively, were obtained. Regarding fleximetry, thermal deformation decreased with an increase in the amount of feldspar substituted with basalt. It was observed that C2 and C3 deformed less at high temperatures than the other combinations of compositions and temperature, probably owing to the lower amount of residual glass phase present during cooling. Compositions with higher substitution amounts of basalt (i.e., C2 and C3) exhibited more stable thermal behaviour than C0.

## 1. Introduction

The ceramic coating market demands large quantities of raw materials, which sometimes entail high logistics expenses. Therefore, the study of alternative materials for application in porcelain compositions has proven to be a current topic, as it can result in a decrease in the use of natural resources, in addition to a possible cost reduction.

Alternative materials have been used in different kinds of clay-based coating materials. Cavallaro et al. [1] investigated the efficacy of several nanoclays (halloysite, sepiolite and laponite) as nanofillers for Mater-Bi, which is a commercial bioplastic extensively used within food packaging applications. They concluded that both the mechanical response to the temperature and the tensile properties make the bio-nanocomposites appropriate for food packaging and smart coating purposes. Abdullah et al. [2] developed a rice husk ash-based geopolymer binder (GB) fire retardant additive (FR) for alkyd paint. They concluded that by developing the optimum rice husk ash-based geopolymer binder for paint formulation, the coating may potentially improve building fire safety through passive fire protection.

According to Zanelli et al. [3], porcelain stoneware tiles are composed of 10–20% clay, 20–30% kaolin, 35–55% feldspar, 0–3% carbonate, 2–5% bentonite, and 0–5% zirconia. Thus, feldspar is the most used item in the formulation, reaching as much as 55% of the porcelain’s composition. One of the main problems of using feldspar, which is an alkaline aluminosilicate, in ceramics is its high cost, owing to the long distances between the feldspar extraction deposits and the ceramic industrial hubs, which are sometimes thousands of km away from the industries. Another disadvantage is that its contaminants can impair the properties of ceramic composition [4].

Feldspars are considered fluxing agents because they provide alkaline oxides, such as Li_2_O, Na_2_O, and K_2_O, which contribute to producing a low-viscosity liquid at high temperatures during firing [4]. Above 1100 °C, they can fill the pores of the ceramic composition by a capillary effect, thus bringing the particles closer together and causing a densification of the ceramic bodies in a process called sintering by viscous liquid phase [5]. This densification depends on, among other factors, the volume and viscosity of the liquid formed [5,6,7,8,9].

Perez [6] studied the influence of lithium, sodium, and potassium feldspars, as well as different combinations between them, on the technological properties of porcelain tiles and concluded that, although this factor cannot only explain the behaviour of porcelain compositions in firing, the compositions containing only nepheline, and those that mix nepheline/feldspar Na:K and dolomite/feldspar Na:K, present acceptable values of fluxing and dimensional stability.

During the production of large plates (size > 0.2 m^2^) in short cycles (~30 min), pyroplastic deformation can occur, which is a dimensional change in ceramic plates, such as porcelain tiles; it occurs during the firing of ceramics owing to the formation of a large amount of low-viscosity liquid phase, whose presence promotes the glazing of the porcelain paste during the cooling process [3,10].

Montedo et al. [11] considered replacing feldspar with a glass-ceramic precursor based on cordierite, which, in addition to generating a liquid of higher viscosity during firing, crystallises during cooling, and thus reduces the pyroplastic porcelain deformation. At 1240 °C, the flexion of the ceramic decreased from 5% for a reference composition (0% feldspar replaced by a cordierite-based glass-ceramic), to 2% in a composition with 100% replacement. In addition, the flexural strength increased from 39.9 (reference) to 70.6 MPa with 100% feldspar replacement by the glass-ceramic. Thus, the possible use of a natural and alternative material to reduce not only the pyroplastic deformation in porcelain compositions, but also the amount of amorphous phase in cooling is worth investigating.

Pazniaki et al. [12] studied the effect of granite and basalt rock residues on the microstructure and properties of porcelain tiles. To this end, they analysed compositions with different levels (up to a maximum of 10%) of feldspar substitution by by-products. Their results showed a decrease in water absorption from 0.4 to 0.2% for samples with substitution levels of 2.5 and 10% at 1200 °C. According to the authors, these results could be attributed to the thermal behaviour of basalt, which is favourable to the formation of a low-viscosity glass phase with a sintering-start temperature in the range of 1150–1160 °C.

Basalt has a dimensional variation of less than 1% when heated to a temperature of 1000 °C in an inert or oxidising atmosphere, which promotes the dimensional stability of ceramic tiles [13]. Thus, it can be deduced that basalt can be used to replace conventional ceramic raw materials and act as a melting material in porcelain products.

In this context, the present work aims at a partial or total replacement of feldspar by basalt to obtain improved porcelain tile compositions. Consequently, a detailed study involving an experimental design of the composition and firing temperature was carried out, specifically focussing on the effect of replacing feldspar with basalt on the pyroplastic behaviour of porcelain bodies.

## 2. Materials and Methods

### 2.1. Materials

The ceramic raw materials used for the specimen manufacturing were supplied by Colorminas Colorífico e Mineração S/A (Criciúma, Brazil). These are raw materials available in the market, and are widely studied and characterised as containing the following: plastic clay (passing through a 325-mesh sieve), quartz (passing through a 325-mesh sieve), sodium potassium feldspar (passing through a 200-mesh sieve), and kaolin (passing through a 325-mesh sieve).

Basalt was obtained from a deposit extraction and processing unit of Sul Brasileira de Mineração Ltda (Urussanga, Brazil). The powder used was a by-product of basaltic rock processing, i.e., obtained from a dry grinding process with the aid of a bag filter. This material had not undergone any type of heating and/or contamination, and retained a relatively fine and uniform particle size, with 80% of the material passing through a 200-mesh sieve (0.074 mm).

### 2.2. Basalt Characterization

The particle size distribution of basalt was evaluated using the laser diffraction method. For this, first, the powders were dispersed in water, using sodium polyacrylate as a dispersing agent and employing the ultrasonic dispersion method. A Cilas PSA 1064 (Orleáns, France) particle size analyser was used. The chemical composition of basalt was determined using X-ray fluorescence (XRF; Panalytical, model Axios Max, Eindhoven, The Netherlands). The structural analysis of basalt was conducted using a Phillips X’Pert MDP X-ray diffraction (XRD, Eindhoven, The Netherlands), with an incident radiation CuKα of 1.5406 Å, reading interval of 5–75° (2θ), step of 0.02°, and step time of 10 s. The thermal behaviour of basalt was determined under a heating microscope, which determined the typical temperatures of melting materials, such as sphere temperature, half-ball temperature, and melting point, by observing the dimensional transformations that occurred in a sample during heating. It is important to know the potential of the material to be used as a flow agent. For this purpose, specific tests were conducted under an optical heating microscope (Expert System Solutions, model Misura HSM ODHT, Modena, Italy), with a heating rate of 40 °C/min in the temperature range of 25–1200 °C.

### 2.3. Effect of Feldspar Substitution by Basalt on Porosity and Flexural Strength

An experimental design with 95% reliability was prepared considering the proportion of feldspar being replaced with basalt and the firing temperature. A 2^2^ factorial design was established, with a central point in duplicate by using the feldspar replacement content by basalt and the firing temperature as the study variables. The independent variable “replacement content” was evaluated between 50 and 100% of feldspar substitution with basalt, while the other independent variable “firing temperature” varied between two levels, defined from the analysis of the linear retraction curve by optical heating microscopy. “Porosity” and “flexural strength” were defined as the response variables.

The reference composition used in this study was defined based on the work of Montedo et al. [11], who used a composition containing 30% plastic clay, 15% kaolin, 10% quartz, and 45% feldspar. The raw materials were dried and mixtures of four compositions were prepared, with C0 being the reference composition (containing 0% basalt, i.e., no replacement); the other three compositions were named C1, C2, and C3, which had 50, 75, and 100% of feldspar replaced with basalt, respectively. The studied compositions are shown in Table 1.

After the drying process in a Cienlab laboratory oven (model CE220/100, São Paulo, Brazil) for 24 h at 100 ± 2 °C, the raw materials were weighed, mixed manually (homogenised), and humidified (7 wt.% water). Subsequently, the compositions were granulated in an ASTM 32-mesh sieve and left to rest in a sealed container.

The chemical constitution of each composition was determined using X-ray fluorescence (XRF; Panalytical, model Axios Max, Eindhoven, The Netherlands). The compositions were formed by uniaxial pressing in a semi-automatic Gabbrielli Technology press (model GT0785, Calenzano, Italy) under a specific pressure of 34.7 MPa. Differential thermal analysis (DTA) was performed using a simultaneous thermal analyser (TA Instruments SDT Q6000, New Castle, USA) at 10 °C/min, up to 1300 °C under air atmosphere (100 mL/min). The firing temperatures of the compositions were determined using optical dilatometry, which measures the dimensional sample variation during heating. This was performed in an optical dilatometer (Expert System Solutions, Misura HSM ODHT, Modena, Italy) at a heating rate of 5 °C/min from 25 to 1250 °C. The specimens used for the dilatometric analysis had approximate dimensions of 13 mm × 5 mm × 5 mm. The temperatures and firing levels were defined for each formulation by considering the maximum linear shrinkage.

Next, the firing of the samples was performed in a Fortelab electric furnace (São Carlos, Brazil), at maximum temperatures ranging between 1160 and 1260 °C, with 6 min of dwell-time at the maximum temperature. Heating rates of 10 °C/min up to 600 °C and 25 °C/min up to the maximum temperature were employed, with the subsequent cooling carried out by thermal inertia of the furnace itself.

A microstructural analysis was performed using a scanning electron microscope (SEM, Zeiss EVO MA10, Cambridge, UK). After cutting and polishing with alumina paste, the specimens were chemically attacked with a 2% solution of hydrofluoric acid, and the samples were covered with gold sputtering (Q156R-ES, Quorum, Lewes, UK).

Crystallographic characterisation of the samples after the firing process was performed by XRD (Phillips X’Pert MDP, Eindhoven, The Netherlands), with CuKα incident radiation (1.5406 Å), and with a reading interval of 5–75° (2θ), step of 0.02°, and step time of 10 s. To capture the results, Inorganic Crystal Structure Database (ICSD) data banks were used. The quantification of the amorphous and crystalline phases was performed using the Rietveld method [14,15,16], combined with an internal standard [17,18,19]. X’pert High Score Plus software (Panalytical, Almelo, The Netherlands) was used to perform the Rietveld refinement. The diffraction was measured using an elemental silicon pattern. The network parameters, grade 3 polynomial background, scale factors, shape of peaks, and unit cells were refined. All the samples subjected to the XRD analyses were mixed with 10% by weight of a fluorite mixture as the internal standard (Xs). Fluorite (CaF_2_) was adopted for this purpose, as it is a stable compound with high purity and good crystallinity, and has a simple crystallographic structure (a cubic system with a few intense diffraction peaks). Equations (1) and (2) were used to calculate the amorphous fraction and proportion of the sample’s crystalline phases, respectively.

(1) $$X_{a}=\left[1-\frac{X_{s}}{X_{SR}}\right]*\left(\frac{1}{1-X_{s}}\right)$$

(2) $$X_{i}=\left[\frac{X_{s}}{X_{SR}}*X_{iR}\;\right]*\left(\frac{1}{1-X_{s}}\right)$$

where *X_a_* is the amorphous phase mass fraction (% *w*/*w*); *X_S_* is the mass fraction of the crystalline phase of the internal pattern (% *m*/*m*); *X_SR_* is the mass fraction of the internal pattern obtained by refinement (% *m*/*m*); *X_i_* is the mass fraction of the crystalline phase analysed (% *m*/*m*); and *X_iR_* is the mass fraction of the analysed phase obtained by refinement (% *m*/*m*).

The porosity was calculated from measurements of apparent density, obtained by the geometric method, and the actual density of the solid, obtained by helium gas pycnometry (Quantachrome pycnometer, Ultrapyc 1200 model, Boynton Beach, FL, USA) using Equation (3). Four samples were used for each of the experimental conditions.

(3) $$P=\left(1-\frac{dap}{dreal}\right)*100$$

where *P* is the porosity (%); d*ap* is the apparent density (g/cm^3^); and d*real* is the actual density of the solid (g/cm^3^).

The water absorption was determined based on C373-18 standard [20]. In short, the sample, kept completely dry at room temperature in a desiccator, is weighed (initial mass, dry weight) and boiled in water for 5 h. After this period of time, the heat source is turned off and the sample is cooled in running water (at room temperature) for 24 h. Then, the excess surface water is removed from the sample with a damp cloth. Then, the sample is weighed (final mass, wet weight). The percentage change in sample mass due to boiling in relation to the initial mass is called water absorption.

The 3-point flexural strength (four samples for each experimental condition; 120 mm × 62 mm × 3 mm specimens; distance of 100 mm between the supports) was measured using a universal mechanical testing machine (EMIC DL10000, São José dos Pinhais, Brazil) with a 10-ton capacity load cell, and a cross-head speed of 1 mm/min.

### 2.4. Effect of Feldspar Substitution by Basalt on Pyroplastic Deformation

Optical fleximetry was used to measure the dimensional variations suffered by the ceramic bodies throughout the firing process; these variations were related to the pyroplastic deformation. The test consisted of an arrangement of a standardised specimen (3 mm × 5 mm × 80 mm) supported on two points. With warm-up, it was expected that this body would form an arrow shape, owing to the effect of gravity [21]. This test was performed on composition samples C0, C1, C2, and C3 in an optical fleximeter (Expert System Solutions, model Misura Flex ODLT, Modena, Italy) at a heating rate of 10 °C/min.

## 3. Results and Discussion

### 3.1. Basalt Characterization

**Figure 1:**
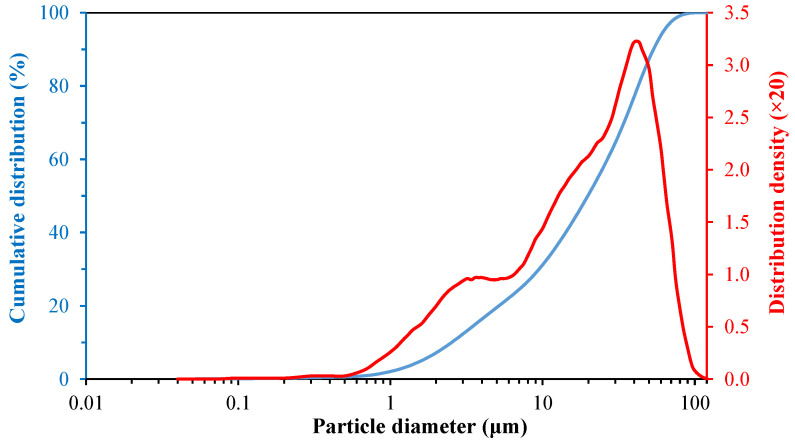
Particle size distribution of basalt.

Figure 1 shows the particle size distribution of basalt, from which a bimodal particle distribution could be observed; one fraction in the size range of 0.2–5 μm with a maximum of 3 μm and another in the size range of 5–100 μm with a maximum of 40 μm were observed. It was also observed that 97% of basalt powder passed through the 200-mesh sieve (0.074 mm).

This distribution was similar to that observed by Dobiszewska and Beycioglu [22], who obtained a bimodal distribution for a basalt powder with particle sizes of 0.5–200 μm, and an average particle size of 20 μm. Barczewski et al. [23] obtained a modal distribution in the range of 3–200 μm, with an average particle size of 40 μm.

**Figure 2:**
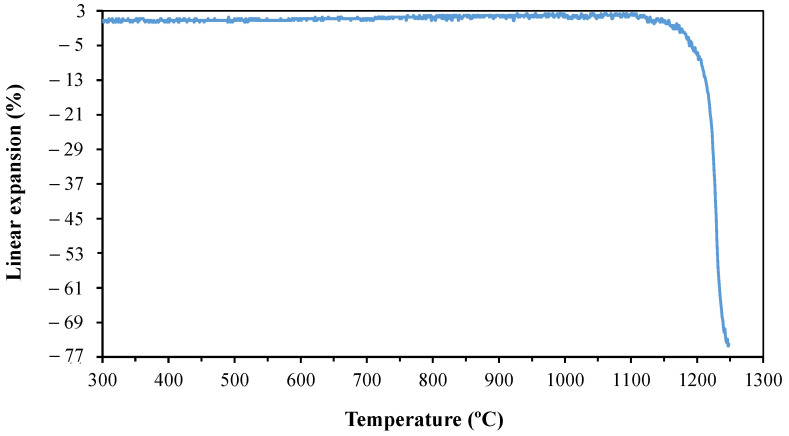
Linear expansion of basalt obtained by heating microscopy.

Figure 2 shows the linear expansion plot of basalt obtained by heating microscopy. Basalt has dimensional stability up to approximately 1100 °C, after which it undergoes the sintering process. The densification begins at approximately 1150 °C.

Frizzo [24] evaluated the retraction curve of feldspar and observed that the retraction began at approximately 1100 °C. Therefore, the viscosity of the liquid formed from the basalt in this study was possibly higher than that of the feldspar studied by Frizzo [24] at the same temperature, because basalt began to undergo the sintering process in this study at approximately 1150 °C.

Table 2 shows the result of the chemical analysis by XRF of basalt, in terms of its oxides. The results presented in Table 2 show that basalt mostly comprises silica (52.01%) and alumina (18.03%) because it is predominantly composed of aluminium silicates, and potassium, sodium, and phosphorus oxides [25].

Other transition metals that are present in most basalts are iron and titanium [13]. Also present in considerable quantities in the studied basalt were the flux elements Fe, Mg, Na, and K, which together added up to 17.27%, which is common in formulations of ceramic masses. Uneík and Kmecová [26] worked with a basalt powder of a similar chemical composition, but containing greater quantities of SiO_2_, along with similar amounts of Al_2_O_3_, CaO, Fe_2_O_3_, MgO, and TiO_2_.

Although it is unusual to find iron oxide in porcelain compositions in significant quantities, it has the characteristics of a melting material and is important in the production of red ceramics [27]. Among the minority components, iron oxide is important because it represents more than 50% of the fluxes present in basalt, that is, 9.35% of the total composition.

The basalt composition is typically 43–47% SiO_2_, 11–13% Al_2_O_3_, 10–12% CaO, and 8–10% MgO, in addition to other oxides that are present at levels below 5% [13]. However, the disadvantage of using iron-rich raw materials is in terms of the colour of the material after firing. According to Roveri et al. [28], at above 1000 °C, the release of bivalent iron occurs, which, by oxidation, becomes trivalent iron, responsible for the red colour; furthermore, at temperatures above 1100 °C, super firing begins to occur and trivalent iron begins to reduce, thus generating a dark reddish-brown to black colour.

**Figure 3:**
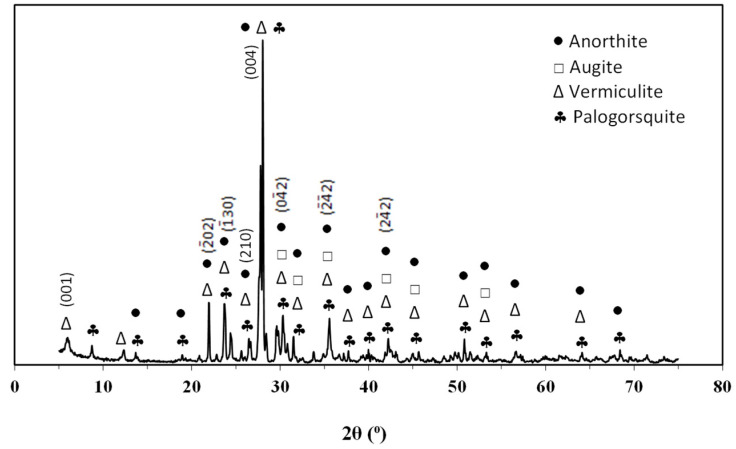
XRD patterns of basalt.

The mineralogical composition of basalt is shown in Figure 3.

The basalt diffraction revealed mostly the presence of anorthite crystalline phase (CaAl_2_Si_2_O_8_, ICSD 63547), one of the plagioclase series minerals and commonly found in igneous and metamorphic rocks; furthermore, the augite phase [(Ca,Na)(Mg,Fe,Al,Ti)Si_2_O_6_, ICSD 75294] was also found. The augite crystalline phase occurs mainly in tabular crystals of basalts and other dark-coloured igneous rocks as it contains iron. It is also a common constituent in lunar basalts and meteorites, rich in basaltic material. The presence of augite explains the high concentration of silicon found in the chemical analysis, as well as the concentrations of iron, aluminium, and sodium. Basalt also contains vermiculite [(MgFe,Al)_3_(Al,Si)_4_O_10_(OH)_2_·4H_2_O, ICSD 034812] and palygorskite [(Mg,Al)_2_Si_4_O_10_(OH)·4H_2_O, ICSD 040687]. According to numerous bibliographies [10,29,30,31], augite is the most common mineralogical phase found in basalts.

### 3.2. Effect of Feldspar Substitution with Basalt on Porosity and Flexural Strength

Table 3 presents, in terms of oxides, the results of the chemical analysis by XRF of the reference formulation C0, as well as the other studied compositions.

As expected, Table 3 shows that the iron content increased with an increase in the proportion of feldspar substitution by basalt, due to its higher Fe_2_O_3_ content. Calcium and magnesium levels also increased, while potassium decreased; sodium remained practically unchanged. Although the content of molten oxides increased with an increase in the proportion of feldspar replaced with basalt, this increase was predominantly of alkaline-earth oxides (Table 4).

According to Novaes [32] and Baucia et al. [33], the proportion of the main fluxes (Fe_2_O_3_ + Na_2_O + K_2_O) varied between 5% and 8% in the studied compositions (Table 4).

Thus, it was expected that the viscosity of the liquid formed during the sintering would be higher, and consequently a reduction in the tendency of pyroplastic deformation of the firing material can be expected.

**Figure 4:**
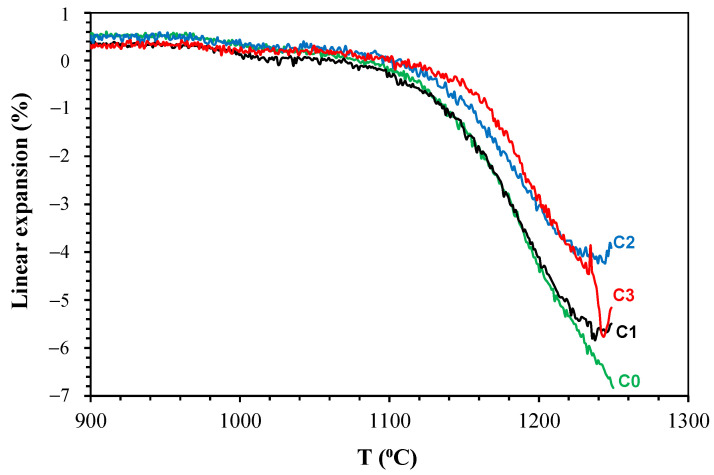
Linear expansion as a function of temperature of the studied compositions.

Figure 4 shows the effect of temperature on the linear expansion of the studied compositions. It can be observed that the compositions began to retract at approximately 980 °C as a result of the sintering process (densification); at 1140 °C, the linear shrinkage began to be accentuated, and the maximum value was reached at different temperatures. The compositions studied expanded from 1240 °C onwards. Firat et al. [34] analysed porcelain compositions with additions of 5–20% of basalt replacing quartz; a linear expansion test showed that samples containing 10, 15, and 20% basalt had sudden shrinkage at 1200 °C. As shown in Figure 4, in this study as well, the replacement of feldspar by basalt revealed a decrease in the linear shrinkage of the samples. Firat et al. [34] also attributed this decrease to the presence of alkaline-earth elements present in basalt, which would form a higher-viscosity liquid phase during the firing. At approximately 1240 °C, the linear shrinkage decreased, and a thermal expansion occurred.

From Figure 4, three suitable firing temperatures were selected within the sintering temperature ranges that were applicable for all the samples tested, namely, 1200, 1220, and 1240 °C. This temperature range was in accordance with the thermal behaviour of basalt, as shown in Figure 2. From the definition of the firing temperatures, it was necessary to define the firing level to achieve the maximum densification of each composition studied. Conducting this test for each of the different samples was extremely important because it ensured that, during the sintering, the samples remained at the maximum sintering temperature for the shortest time required. It also helped in energy-saving by avoiding longer firing times at maximum temperatures.

**Figure 5:**
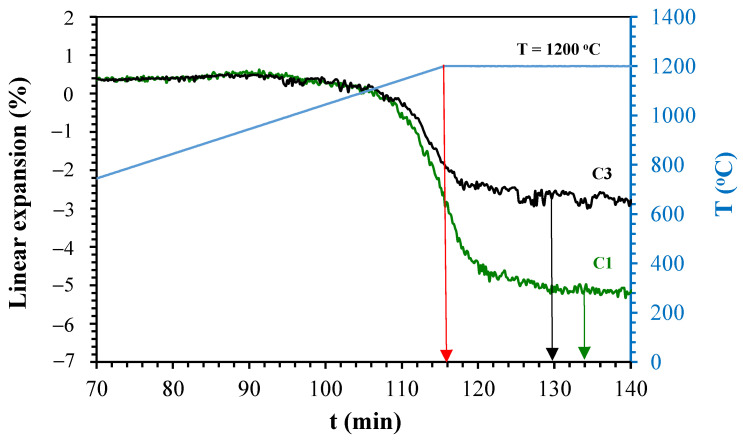
Linear expansion as a function of holding time of C1 and C3 at 1200 °C.

**Figure 6:**
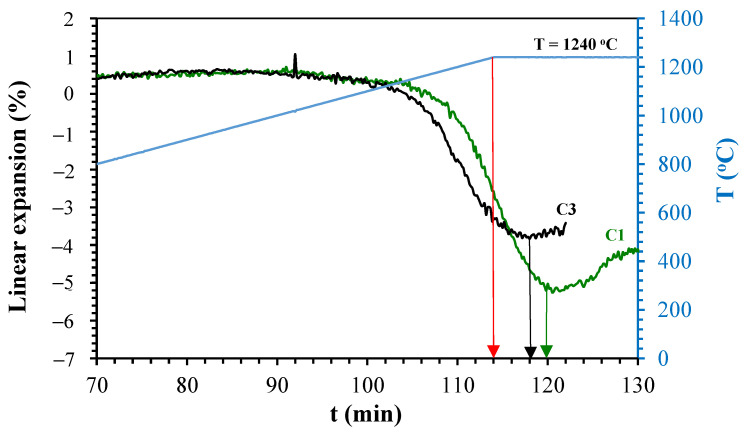
Linear expansion as a function of holding time of C1 and C3 at 1240 °C.

**Figure 7:**
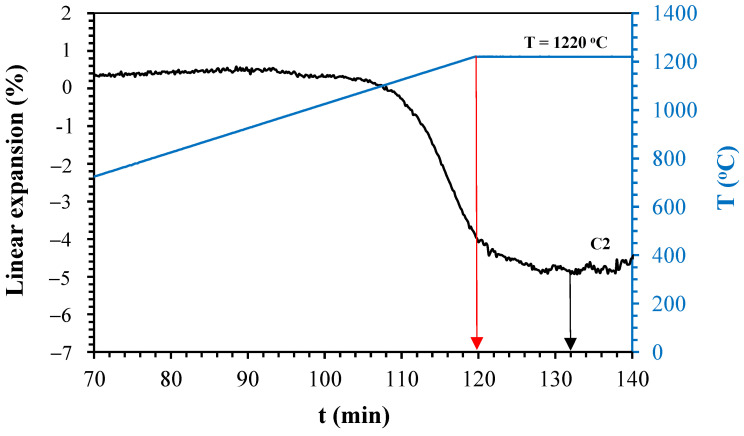
Linear expansion as a function of holding time of C2 at 1220 °C.

**Figure 8:**
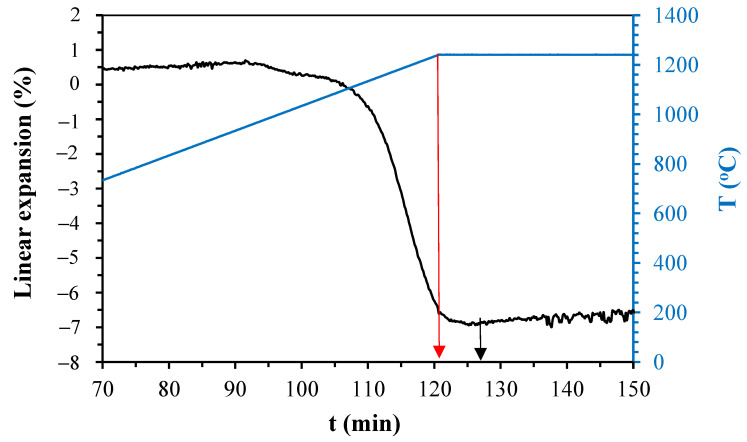
Linear expansion as a function of holding time of C0 at 1220 °C.

Figure 5, Figure 6, Figure 7 and Figure 8 show the linear expansion curves as a function of the firing level for each composition studied. In the graphs, the red arrows indicate the beginning of the sintering level at the maximum temperature; on the other hand, the black and green arrows indicate the final time, at which the samples remained at the maximum temperature.

From Figure 5, Figure 6, Figure 7 and Figure 8, the temperature levels chosen for the studied compositions were selected (summarised in Table 5). These temperatures and firing levels were used to process the samples for characterisation.

**Figure 9:**
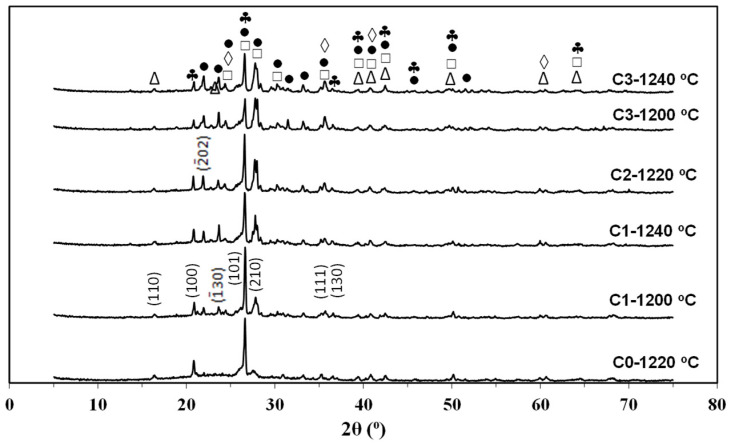
XRD patterns of studied compositions: ◊ Hematite; ♣ Quartz; ∆ Mullite; □ Augite; • Anorthite.

The X-ray diffractograms of all the compositions (C0, C1, C2, and C3), and the firing temperatures studied are shown in Figure 9.

The diffractograms show the presence of crystalline phases mullite (ICSD 75305), augite (ICSD 75294), quartz (ICSD 31228), and anorthite (ICSD 63547); hematite (Fe_2_O_3_, ICSD 33643) was also present in small quantities with low-intensity peaks.

There are more intense peaks of anorthite (ICSD 63547) in C1, C2, and C3, which are common in ceramic compositions containing igneous rocks, such as feldspar. Quartz was present in all compositions, with C0 having the most intense peaks. Augite was not present in C0; however, it was present in all the compositions that used basalt.

Hematite was identified only in C2 and C3 because of the greater amount of basalt added in them. Mullite was present in all the studied compositions, and its content varied according to the formulation and firing temperature. According to Zanelli et al. [3], mullite and quartz phases are present in most ceramic formulations, followed by different mineralogical phases depending on the raw materials and materials used in their manufacture [35,36].

Table 6 shows the results of phase quantification using Rietveld refinement of the compositions studied. It was observed that the levels of the anorthite and augite phases increased with an increase in the basalt content at the same temperature, as these were the main crystalline phases found in pure basalt; however, they decrease with an increase in the temperature under the same basalt content. Moreover, their quartz levels decreased compared to that in C0.

The amorphous phase was quantified using the internal standard method. Table 6 also shows the effect of replacing feldspar by basalt in the residual amorphous phase. As expected, this substitution promoted the formation of a smaller amount of the residual amorphous phase during the firing process. Iron acts as a flux during the sintering [27]; however, it forms crystalline phases during the cooling stage, such as hematite in small quantities. The amorphous phase decreased with the increase in the basalt content and the temperature decrease. This reduction in the amount of residual amorphous phase, especially the vitreous phase, had a direct impact on pyroplastic deformation, as will be seen below.

Table 6 also shows the values of the weighted index (Rwp) and the adequacy of the refinement adjustment index (goodness of fit; GOF). GOF demonstrates the level of quality of a refinement, and whose values close to 1 represent optimal levels that have already reached their expected limiting value for the measured diffraction data [37]. A value of approximately 5 can be considered satisfactory. Rwp, on the other hand, represents the weighted index/error. From a mathematical point of view, it is one of the indices that best reflects the progress of refinement because it minimises the residue in the numerator [38]. Although the Rwp and GOF values are slightly high, Kinast [39] claims that it is important to know that the fit quality values are purely numerical and do not reflect the quality of a good fit. Therefore, user sensitivity is important in visual analysis, i.e., in verifying whether the peaks proposed by the model resemble the experimental diffraction.

**Figure 10:**
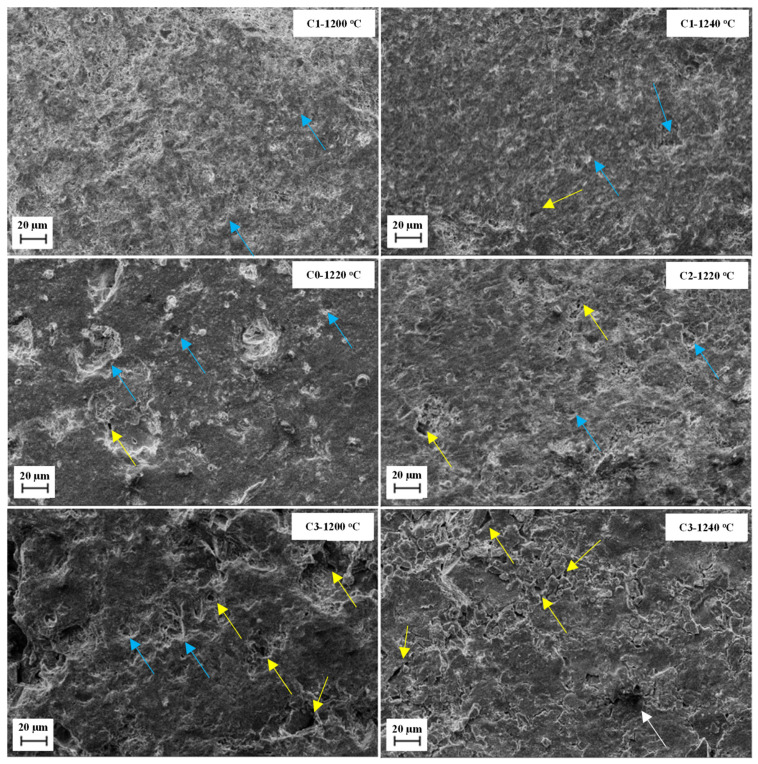
Micrographs (SEM) of studied compositions with specimens fractured after firing. Yellow arrows indicate open pores and blue arrows indicate closed pores.

Figure 10 shows the microstructures obtained by SEM (500× magnification) of the studied samples after firing (on the fracture face). It can be observed that the samples have very similar microstructures. However, the samples of C3 (i.e., samples with the highest basalt content) are more porous. In addition, the existence of some closed pores can also be observed. It is plausible that, owing to the regularity of the pores and the levels of the formed crystalline phases (Table 6), the flexural strength increased in relation to the reference sample, even with an increase in the porosity.

Supawan et al. [40] explained, with the use of basalt as a raw material for ceramic production, that the addition of basalt increased the pore formation at temperatures above 1200 °C. They suggested that this phenomenon would result from secondary melting agents, such as CaO and MgO present in basalt, and that these oxides can increase or accelerate the liquid-phase densification. Pazniak et al. [12] explained that the main mechanism that occurred during porcelain firing was the formation of the liquid-phase, wherein the degree of sintering was associated with the amount of molten mass present. Basalt favours the beginning of sintering by liquid-phase formation at temperatures below 1150 °C and consequently the formation of pores.

Table 7 shows the porosity results of the compositions studied. Although the results were statistically equivalent for C1, C2, and C3, there was a tendency of porosity increasing with an increase in the feldspar replacement content by basalt, and also with the temperature increase.

Table 8 shows the analysis of the porosity value variance. Even though firing temperature had the highest values of F and p, and consequently had the greatest relevance to porosity values, its statistical significance was zero because the R2 value was 0.0 [41].

The reliability observed by the *p*-value was also low (82%). Thus, the analysis of variance demonstrated that the firing temperature was the factor with the greatest influence on porosity. The lowest values were obtained for C1 at 1200 °C (19.3 ± 2.8%) and for C2 at 1220 °C (17.7 ± 3.7%). All the formulations with the basalt addition showed greater porosity than the reference formulation (C0).

An increase in porosity could be observed with an increase in the firing temperature. Pazniak et al. [12] explained that this was owing to the increase in the iron oxide content in basalt compositions, which promotes the over-firing effect. However, some of these pores were closed, as observed in the micrographs in Figure 10.

However, this behaviour of porosity as a function of composition and firing temperature was not observed in water absorption, because the over-firing of the porosity is normally close. Table 9 shows the water absorption results of each composition studied as a function of the firing temperature. Unlike porosity, the water absorption decreased with increasing firing temperature, which means that the number of open pores decreased, and the number of closed pores increased.

It should be noted that for C1, the water absorption decreased with an increase in the firing temperature, as expected because there was a greater liquid-phase formation during the sintering, and therefore a greater densification. 

For C3, however, it is clear that for both tested temperatures, the water absorption did not differ. The lowest water absorption value, i.e., 2.7 ± 0.4%, was obtained for C1 at 1240 °C.

Pazniak et al. [12] explained that the main mechanism that occurs during porcelain firing is the formation of a liquid phase, wherein the degree of sintering is associated with the amount of molten mass present. Basalt favours the beginning of sintering via a liquid-phase formation at temperatures below 1150 °C and through pore formation.

Table 10 shows the flexural strength results of each studied formulation as a function of firing temperature. For both C1 and C3, there was an increase in the flexural strength with an increase in the firing temperature. C1 showed a greater flexural strength up to 1240 °C, and at the same time, greater porosity and lower water absorption, thus fitting into the classification of porcelain stoneware according to the ceramic coating standard NBR 13818 [42]. It can be observed that at 1200 °C, the increase in the content of feldspar substitution by basalt (from C1 to C3) increased the flexural strength. However, at 1240 °C, the effect was reversed, probably because the open porosity increased from C1 to C3.

Previous studies using basalt in ceramic compositions yielded very different flexural strength values. Supawan et al. [40] obtained flexural strength values between 15 and 118 MPa; their formulation contained 20% basalt, which was fired at 1200 °C, and obtained a flexural strength of 23 MPa—a value similar to that obtained in this study for C1 fired at 1240 °C. Njindam et al. [43] produced porcelain stoneware with the addition of a glass powder residue, and obtained flexural strength values between 8 and 38 MPa. The composition was fired at 1150 °C containing 20% glass powder and reached a resistance of 23 MPa.

Table 11 shows the analysis of variance results for the flexural strength of the studied compositions. The firing temperature had the highest values of F and p; therefore, it had the greatest relevance for the flexural strength, and a statistical reliability very close to 100%.

Another result that achieved a high reliability (98%), but with lower significance than the firing temperature, was the interaction between the composition and firing temperature; therefore, it was concluded that, in addition to the firing temperature, the interaction between the two factors had a strong relevance to the flexural strength. The R2 and the R2-adjusted values were 99.3 and 99.4, respectively, thus indicating a strong statistical reliability [41].

**Figure 11:**
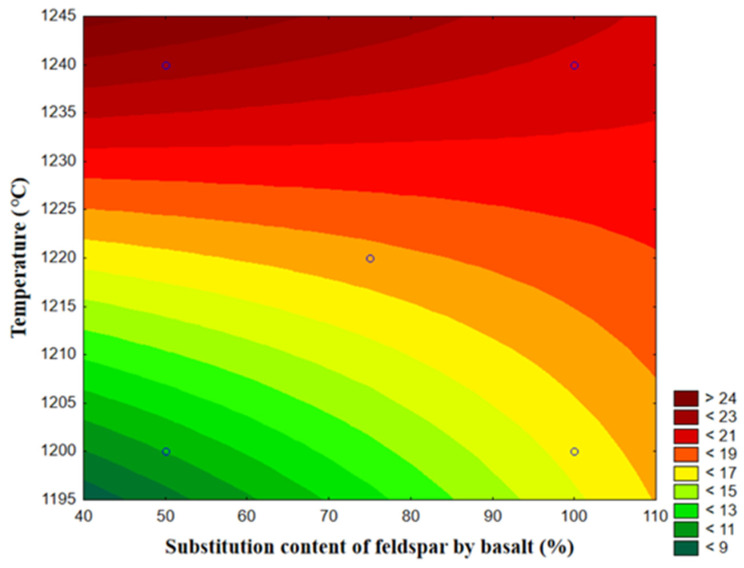
Response surface of the samples related to the flexure strength of studied compositions.

Figure 11 shows a graph of the response surface of the samples related to the flexural strength, considering the content of feldspar substitution by basalt as a function of the firing temperature. 

The area with the highest firing temperature and lowest replacement content had the highest flexural strength.

Considering the results presented above, it is clear that the firing temperature had a significant effect on the flexural strength, according to the results in Table 11, while the proportion of feldspar replaced by basalt had less influence. Although statistically identical, the porosity results indicated an increase with an increase in firing temperature. The same was true of the flexural strength.

### 3.3. Effect of Feldspar Substitution with Basalt on Pyroplastic Deformation

**Figure 12:**
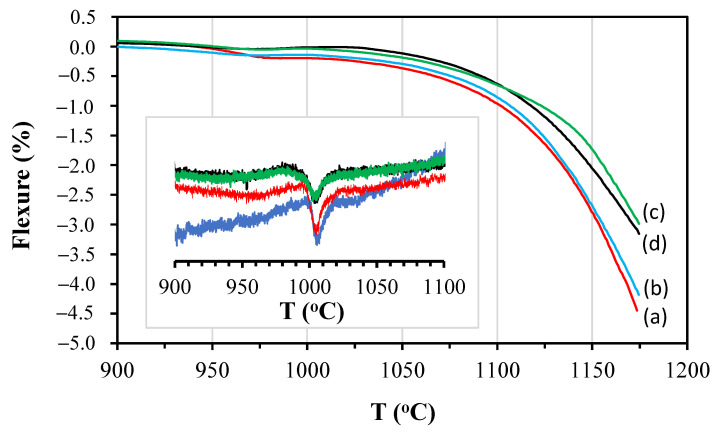
Optical fleximetry of studied compositions: (a) C0; (b) C1; (c) C2; (d) C3. Highlighted DSC plot: C0—blue line; C1—red line; C2—black line; C3—green line.

Figure 12 shows the optical fleximetry results of the studied compositions; in addition, it also shows the differential scanning calorimetry (DSC) results. Pyroplastic deformation occurs when, during firing, a viscous liquid phase is formed, which causes the formation of a concavity owing to gravity. This phenomenon is common in high vitrification products and, for this reason, attention should be paid to deformations of ceramic bodies [10]. During cooling, the fluid formed solidifies and the dimensions of the body become practically permanent. Thus, the partial crystallisation of the liquid phase could reduce this effect.

It can be observed that, approximately, from a temperature of 900 °C, the studied compositions began to show a negative deformation, i.e., concavity, owing to the beginning of the formation of a liquid phase from the fusion of clay minerals and the action of gravity [21]. Between 975 and 1025 °C, the increase in the deformation was interrupted by the formation of mullite at 1005 °C, which stabilised the structure, as shown in the DSC graph of the studied compositions in Figure 12. From approximately 1025 °C, the deformation increased with increasing temperature, and was lower for higher levels of feldspar substitution with basalt.

To ensure safety during the tests, the maximum temperature was limited to 1170 °C. Thus, to correlate the temperatures studied (1200, 1220, and 1240 °C), the temperature differences were maintained, but only in the temperature measurement range of fleximetry, i.e., 1130, 1150, and 1170 °C. Table 12 shows the flexion values obtained by optical fleximetry at the defined temperatures of the studied compositions. 

The results show that the flexion increased with an increase in temperature, with a reduction in the content of feldspar substitution by basalt, and with an increase in the amorphous phase content (see also Table 6).

C2 and C3, with higher basalt contents, showed lower pyroplastic deformation (–3.0 and –3.2%, respectively) at 1150 and 1130 °C, respectively, probably owing to the formation of a greater amount of crystalline phases, as observed in the quantification by the Rietveld method (Table 6).

Thus, a direct relationship between the pyroplastic deformation and residual amorphous phase content of the porcelain composition firing process was obvious. A reduction in the amorphous phase content was obtained with higher levels of feldspar substitution by basalt. However, to achieve higher densification levels, the firing temperature should be increased, which would increase the pyroplastic deformation. To overcome this, thinner powders (lower D50) of basalt could be used, which allow sintering at lower temperatures for the same desired water absorption, but reduce the pyroplastic deformation.

Considering the results obtained, C1 fired at 1240 °C showed water absorption that classifies the composition as porcelain, yielded higher flexural strength, and showed flexion (pyroplastic deformation) lower than that of C0. C2 fired at 1220 °C could also be used; however, for this, it may be necessary to use a basalt with a smaller average particle size.

## 4. Conclusions

A study to evaluate the effects of feldspar replacement with basalt in porcelain tile composition was carried out, especially with regard to pyroplastic deformation. The porosity increased with increasing temperature and with the increase of the feldspar content replaced by basalt. The porosity of composition C1 (50% replacement) increased with the temperature increasing. Composition C3 (100% replacement) at 1200 °C showed a porosity of 21.3 ± 3.4%. Thus, it can be concluded that the content of feldspar substituted with basalt directly influenced the porosity, which was statistically more significant than the firing temperature. The same was observed for the flexural strength. C3 showed a flexural strength of 15.3 ± 2.0 MPa at 1200 °C. Except for C1 at 1200 °C, all other experimental conditions showed flexural strength greater than C0 (0% replacement). Analysis of result variance indicated that both independent variables (firing temperature and feldspar content replaced by basalt) influenced the porosity and flexural strength; however, the firing temperature had a greater influence than the feldspar content substituted with basalt. C1 up to 1240 °C showed water absorption of 2.7 ± 0.4% and can be classified according to NBR 13818 as porcelain stoneware. C2 and C3, with higher basalt levels, showed lower pyroplastic deformations (–3.0 and –3.2%, respectively) at 1150 and 1130 °C, respectively, probably due to the formation of a greater number of crystalline phases. Considering the results obtained, C1 fired at 1240 °C showed water absorption that classifies the composition as porcelain and possessed higher flexural strength; however, its flexion (pyroplastic deformation) was lower than that of C0. C2 fired at 1220 °C could also be used, but with a smaller basalt particle size.

## Figures and Tables

**Table 1:** Compositions studied (wt.%).

Material	C0	C1	C2	C3
Clay	40	40	40	40
Feldspar	45	22.5	11.25	0
Basalt	0	22.5	33.75	45
Kaolin	12	12	12	12
Quartz	3	3	3	3

**Table 2:** Chemical analysis by XRF of basalt.

Chemical Compound	Content (wt.%)
SiO_2_	52.01
Al_2_O_3_	18.03
CaO	10.90
Fe_2_O_3_	9.35
MgO	4.08
Na_2_O	3.14
TiO_2_	1.28
K_2_O	0.70
P_2_O_5_	0.30
MnO	0.14
Loss on ignition	0.08

**Table 3:** Chemical composition by XRF of studied compositions.

Chemical Compound (wt.%)	Studied Compositions			
	C0	C1	C2	C3
SiO_2_	62.51	57.66	55.49	54.54
Al_2_O_3_	22.32	22.71	22.85	22.67
CaO	1.34	3.91	4.87	6.10
Fe_2_O_3_	0.83	3.05	4.16	5.14
MgO	0.41	1.37	1.76	2.19
Na_2_O	1.96	1.89	1.97	1.85
TiO_2_	0.12	0.41	0.56	0.70
K_2_O	5.00	2.95	2.09	1.04
P_2_O_5_	0.04	0.09	0.12	0.15
MnO	0.03	0.07	0.08	0.09
Loss on ignition	5.44	5.90	6.05	5.53

**Table 4:** Flux agent composition by XRF of the studied compositions.

Flux Agent (wt.%)	Studied Compositions			
	C0	C1	C2	C3
Fe_2_O_3_	0.83	3.05	4.16	5.14
Na_2_O	1.96	1.89	1.97	1.85
K_2_O	5.00	2.95	2.09	1.04
CaO	1.34	3.91	4.87	6.10
MgO	0.41	1.37	1.76	2.19
Total	9.54	13.17	14.85	16.32
Na_2_O + K_2_O	6.96	4.84	4.06	2.89

**Table 5:** Holding times at different firing temperatures of the studied compositions.

Studied Composition	Holding Time (min)		
	1200 °C	1220 °C	1240 °C
C0		6	
C1	12		7
C2		12	
C3	20		4

**Table 6:** Phase quantification of the studied compositions obtained by Rietveld refinement.

Phase Quantification (%)							
Crystalline Phase	Chart (ICSD)	C0	C1-T1	C1-T3	C2-T2	C3-T1	C3-T3
Anorthite	63547	4.3	28.3	25.2	26.6	40.2	29.1
Augite	75294	0	4.0	0.3	2.7	3.5	5.6
Quartz	31228	12.5	7.9	6.6	7.0	7.5	5.6
Mullite	75305	3.5	2.5	4.5	3.1	4.8	4.4
Hematite	33643				0.7	1.9	0.7
Amorphous		79.7	57.3	63.4	60.0	42.0	54.6
R_WP_		32.1	20.6	23.3	24.3	24.4	22.2
GOF		13.7	15.3	18.4	8.2	21.4	20.7

Where Rwp is the weighted index and *goodness of fit* (GOF) is the adequacy of the refinement adjustment.

**Table 7:** Porosity of studied compositions at different temperatures.

Composition	Porosity (%)		
	1200 °C	1220 °C	1240 °C
C1	19.3 ± 2.8		22.6 ± 1.4
C2 (1)		20.1 ± 3.1	
C2 (2)		17.7 ± 3.7	
C3	21.3 ± 3.4		22.2 ± 0.7
C0		15.0 ± 7.9	

**Table 8:** Analysis of the porosity values variance for the studied compositions.

	SS	Df	ms	F	p
Composition (1)	4.18	1	4.18	0.36	0.55
Firing temperature (2)	22.54	1	22.54	1.96	0.18
Interaction between 1 and 2	3.81	1	3.81	0.33	0.57
Error	229.72	20	11.49		
Total SS	260.24	23			

Where F is the association of the response term; SS is the sum of the squares; Df is the variance (freedom levels); ms is the quadratic average; and p is the evidence probability of the null hypothesis.

**Table 9:** Water absorption of studied compositions at different firing temperatures.

Composition	Water Absorption (%)		
	1200 °C	1220 °C	1240 °C
C1	5.1 ± 0.8		2.7 ± 0.4
C2 (1)		4.8 ± 0.9	
C2 (2)		5.6 ± 0.5	
C3	9.0 ± 1.0		8.6 ± 0.9
C0		4.4 ± 0.2	

**Table 10:** Flexure strength of studied compositions at different firing temperatures.

Composition	Flexure Strength (MPa)		
	1200 °C	1220 °C	1240 °C
C1	11.1 ± 0.6		22.2 ± 1.9
C2 (1)		19.5 ± 2.3	
C2 (2)		20.1 ± 1.5	
C3	15.3 ± 2.0		15.8 ± 3.4
C0	13.7 ± 2.2		

**Table 11:** Analysis of variance of flexure strength of studied compositions.

	SS	Df	ms	F	p
Composition (1)	13.45	1	13.45	1.74	0.20
Firing temperature (2)	250.83	1	250.83	32.51	0.00
Interaction between 1 and 2	49.39	1	49.39	6.40	0.02
Error	154.29	20	7.71
Total SS	467.96	23			

**Table 12:** Flexure and amorphous phase content (Table 6) of studied compositions at different temperatures.

Composition	Flexure (%) [Amorphous Phase Content (%)]		
	1130 °C	1150 °C	1170 °C
C1	−1.7 [57.3]		−3.9 [63.4]
C2		−1.8 [60.0]	
C3	−1.3 [42.0]		−3.0 [54.6]
C0	−4.2 [79.7]		

## Data Availability

Not applicable.
